# Depletion of Luminal Pyridine Nucleotides in the Endoplasmic Reticulum Activates Autophagy with the Involvement of mTOR Pathway

**DOI:** 10.1155/2013/942431

**Published:** 2013-11-17

**Authors:** Orsolya Kapuy, Gábor Bánhegyi

**Affiliations:** Department of Medical Chemistry, Molecular Biology and Pathobiochemistry, Semmelweis University, Tűzoltó utca 37-47, Budapest 1094, Hungary

## Abstract

It has been recently shown that redox imbalance of luminal pyridine nucleotides in the endoplasmic reticulum (ER) together with oxidative stress results in the activation of autophagy. In the present study we demonstrated that decrease of luminal NADPH/NADP^+^ ratio alone by metyrapone was sufficient to promote the mechanism of “self-eating” detected by the activation of LC3. Depletion of luminal NADPH had also significant effect on the key proteins of mTOR pathway, which got inactivated by dephosphorylation. These findings were also confirmed by silencing the proteins (glucose-6-phosphate transporter and hexose-6-phosphate dehydrogenase) responsible for NADPH generation in the ER lumen. However, silencing the key components and addition of metyrapone had different effects on downstream substrates 4EBP1 and p70S6K of mTOR. The applied treatments did not compromise the viability of the cells. Our data suggest that ER stress caused by luminal NADPH depletion activates a pro-survival autophagic mechanism firmly coupled to the activation of mTOR pathway.

## 1. Introduction

The endoplasmic reticulum (ER) is a eukaryotic cellular component that acts as an essential integrator of external and internal stimuli by keeping the balance of protein level (so called proteostasis) [1, 2]. ER has a crucial role in folding and secretion of secreted and membrane proteins, calcium storage, and lipid biosynthesis [2, 3]. Recent experimental data have shown that for the proper function of the ER, a high luminal NADPH/NADP^+^ ratio is essential [4, 5]. The reduced luminal NADPH is required for the prereceptorial activation of glucocorticoids (such as cortisol), for some reactions of biotransformation and presumably for local antioxidant defense. NADPH level is primarily sustained by the coordinated action of the ER glucose-6-phosphate transporter (G6PT) and the luminal hexose-6-phosphate dehydrogenase (H6PDH), while NADPH can be consumed by different luminal reductases (such as carbonyl reductases) [6–8]. 

The imbalance of NADPH/NADP^+^ ratio or depletion of luminal NADPH level sensitizes the ER to different oxidative injuries [5]. Starvation has been reported to cause a shift in the redox state of luminal pyridine nucleotides toward the oxidized direction [9]. Experimentally, addition of G6PT blockers S3483 or chlorogenic acid or the ROS generating menadione results in the decrease of luminal NADPH [5, 10]. Metyrapone decreases luminal NADPH level by the stimulation of carbonyl reductases and by carbonyl reductase independent mechanisms [4, 11]. Interestingly, combined treatment of NADPH depleting agents (e.g., inhibition or silencing G6PT, silencing H6PDH, and oxidation of ER luminal pyridine nucleotides) and oxidative stress induced by menadione results in the induction of autophagy markers [5].

It has been also proved that autophagosome formation gets immediately induced in the presence of ER stressors (such as thapsigargin and tunicamycin) [12, 13]. This observation is confirmed by increasing autophagic function. Any disturbance of autophagy accelerates cell death claiming that autophagy plays important roles in cell survival during ER stress [12, 13].

The autophagy plays an important role to “digest” the damaged cytoplasmic components of cells at physiological conditions [14]. In addition autophagy has an essential role in promoting cellular-survival during starvation by “self-eating” of parts of the cytoplasm and intracellular organelles [14, 15]. Autophagy induced by nutrient deprivation can be mimicked by addition of drug rapamycin [16].

Mammalian target of rapamycin (mTOR) alias FKBP-rapamycin associated protein (FRAP) is an evolutionally conserved serine/threonine kinase of mTOR pathway that is a major effector of cell growth and proliferation by controlling protein synthesis [17, 18]. The phosphorylated form of mTOR/FRAP is active, while the kinase becomes immediately inactive and dephosphorylated upon nutrient depletion [18]. Some of the targets of mTOR/FRAP are phosphorylated directly, such as Eukaryotic translation initiation factor 4E-binding protein 1 (4EBP1) and Ribosomal protein S6 kinase 1 (p70S6K). Both 4EBP1 and p70S6K become dephosphorylated in response to starvation resulting in block of protein translation [18, 19]. Some new results have shown that DNA damage and ER stress-related Gadd34 is also required for autophagy induction under nutrient-depleted conditions [20]. The Gadd34 knock-downed mice cannot promote starvation-dependent mTOR/FRAP kinase dephosphorylation [21]. However, beside CHOP/Gadd153, Gadd34 is also a crucial member of unfolded protein response (UPR) [13]. UPR is a complex network of signalling pathways measuring both the not properly folded protein level and the precise balance between production and consumption of folded proteins [13, 22]. 

In this study we reexamined the role of metyrapone in autophagy induction claiming that NADPH depletion without combination of oxidative agent can be sufficient to enhance autophagic process. Treatment with NADPH depleting agents results in dephosphorylation of the key molecules of mTOR pathway confirming that the imbalance of ER luminal pyridine nucleotides induces a starvation phenotype.

## 2. Materials and Methods

### 2.1. Materials

Metyrapone, rapamycin, 3-methyladenine, Wortmannin, and trypan blue were purchased from Sigma. All other chemicals were of reagent grade.

### 2.2. Cell Culture and Maintenance

As model system, human liver carcinoma (HepG2) cell line was used. Due to the relatively large dimension of ER in HepG2 cells they offer an excellently suitable *in vitro* model system for studying the effect of addition of different type of ER stressors. HepG2 cells were maintained in DMEM medium supplemented with 10% fetal bovine serum and 1% antibiotics/antimycotics. Culture dishes and cell treatment plates were kept in a humidified incubator at 37°C in 95% air and 5% CO_2_.

### 2.3. RNA Interference

The silencing of G6PT and H6PDH was managed by transfection with siG6PT and/or siH6PDH, respectively. The corresponding RNA interference was performed using Lipofectamine RNAi Max (Invitrogen) in GIBCOTM Opti-MEM I (GlutaMAXTM-I) Reduced-Serum Medium liquid (Invitrogen) and 20 pmol/mL siRNA. Interfering RNAs for human G6PT (gene ID: NM 001467) and H6PD were constructed by Invitrogen. The used oligonucleotide for human G6PT: 5′-CGAAACAUCCGCACCAAGAdTdT-3′ (sense) and 5′-UCUUGGUGCGGAUGUUUCGdTdT-3′ (antisense) [23]. The used oligonucleotide for human H6PD was 5′-UUAUGGAGACAUGUCCCUGGAGCUC-3′ (sense) and 5′-GAGCUCAGGGACAUGUCUCCAUAA-3′ (antisense). Interfering materials were annealed duplexes in both cases [5].

### 2.4. SDS-PAGE and Western Blot Analysis

HepG2 cells were harvested and lysed with 20 mM Tris, 135 mM NaCl, 10% glycerol, and 1% NP40 at pH 6.8. Protein content of cell lysates was measured using Pierce BCA Protein Assay. Equal amounts of protein were used in each procedure. SDS-PAGE was performed by using Hoefer miniVE (Amersham). The protein patterns were transferred onto Millipore 0.45 *μ*m PVDF membrane. Immunoblotting was accomplished with TBS Tween (0.1%), containing 5% nonfat dry milk for blocking membrane and for antibody solutions. The correct loading was controlled by staining with Ponceau S in each experiment. The following antibodies were used: antiGADD153, antiLC3B, antiGADD34, antimTOR/FRAP, antimTOR/FRAP-P, antip70S6K, antip70S6K-P, antiprocaspase 3, antiGAPDH (SantaCruz), anti4EBP1, anti4EBP1-P, antiPARP, antieIF2alpha-P, antieIF2alpha (Cell Signaling), and HRP conjugated secondary antibodies (SantaCruz).

### 2.5. Cell Viability Assays

The number of viable cells was detected using a trypan blue exclusion assay. Cells were incubated with isotonic solution of trypan blue 0.6% and both permeabilized and nonpermeabilized cells were counted. In case of each experiment at least three parallels were measured. Cell viability was also determined by CellTiter-Blue Assay from Promega. 20 *μ*L of reagent was added to each 100 *μ*L of medium in a 96-well plate. In this method the viable cells are able to reduce the indicator dye resazurin into resorufin, while the nonviable cells lose their metabolic capacity. The absorbance was read at 570 nm using 600 nm as a reference wavelength.

### 2.6. HPLC Detection of Metyrapone

The uptake of drug metyrapone was measured via HPLC. Cell cultures were incubated for 0, 10, 20, 30, 40, and 120 min, then cells and media were separated, and cells were lysed with 20 mM Tris, 135 mM NaCl, 10% glycerol, and 1% NP40 at pH 6.8. 150 *μ*L media of each sample was directly added to 150 *μ*L ice-cold methanol. The protein concentration of each sample was set to 0.075 mg/mL with MOPS buffer. Then 150 *μ*L of these samples were added to 150 *μ*L ice-cold methanol. Samples were stored at −20°C until analysis. After sedimentation of the precipitates by centrifugation (20 000 ×g for 10 min at 4°C), the metyrapone content of the supernatants was measured by HPLC (Alliance 2690; Waters Corp., Milford, MA, USA) using a Nucleosil 100 C18 column (5 *μ*m 25 × 0.46) (Teknokroma). The eluent was 58% methanol, samples were eluted for 30 min and the absorbance was detected at 245 nm wavelength (Dual l Absorbance Detector 2487). The retention times of metyrapone (approx. 15 min) was determined by injecting standards.

## 3. Results 

### 3.1. NADPH Depletion in the ER Lumen by Metyrapone Results in Autophagy Activation and Downregulation of MTOR Pathway

**Figure 1:**
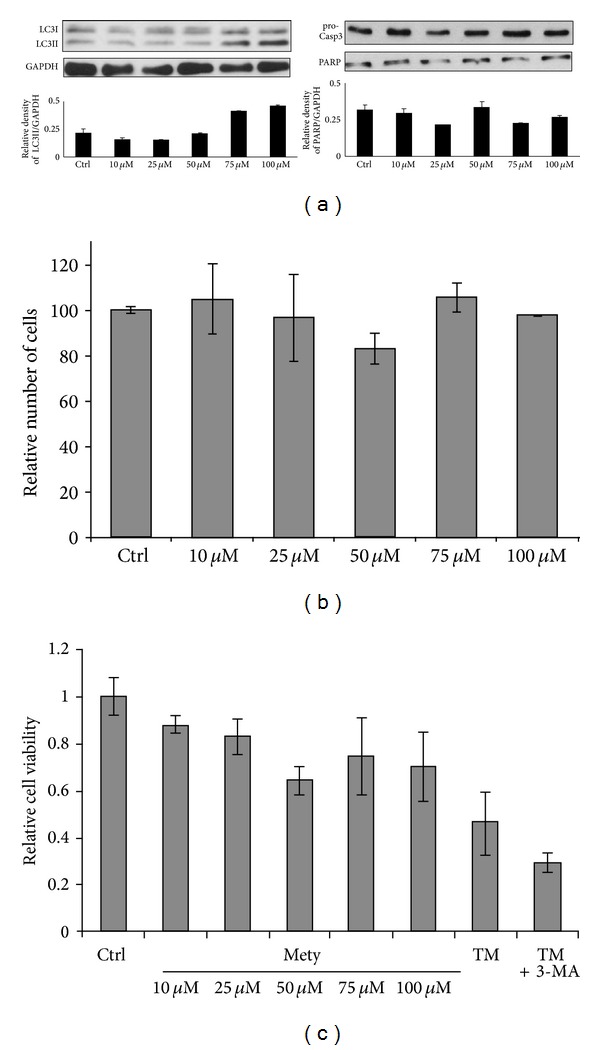
NADPH depletion induced by metyrapone is sufficient to promote autophagy. HepG2 cells were treated with different amount of metyrapone (10 *μ*M–100 *μ*M) for 1 h at 37°C. (a) Cell lysates were analysed by Western blot by immunoreacting with antibodies against LC3, procaspase 3, PARP, and GAPDH, respectively. The relative density of both the lower band of LC3/GAPDH and PARP/GAPDH were plotted. (b) Cell viability was assessed by counting the cells both permeable and nonpermeable for trypan blue. About 95% of the cells were nonpermeable for trypan blue. (c) Cell viability was followed by using Cell Titer-Blue Assay and the relative cell viability was represented after 1-hour long metyrapone treatment. As positive controls cells were treated with tunicamycin (TM—25 *μ*M) for 2 hours combined with/without 2-hour long 3-methyladenine pretreatment (3-MA—10 mM).

We have shown previously that combination of depletion of ER luminal NADPH and menadione-induced oxidative stress injury results in autophagy and decreased viability in HepG2 cells [5]. However, under experimental conditions performed in those experiments imbalance of NADPH level caused by metyrapone alone was not sufficient to induce autophagic events or viability alterations. Here we found that prolonged incubation was not sufficient to induce autophagy (data not shown), but the increased concentration of metyrapone (75–100 *μ*M) was able to promote formation of autophagy marker, LC3II; however, the apoptosis markers, such as cleavage of PARP and procaspase 3, did not show any apoptotic event suggesting that an autophagy-dependent process switches on during metyrapone treatment (Figure 1(a)). 

**Figure 2:**
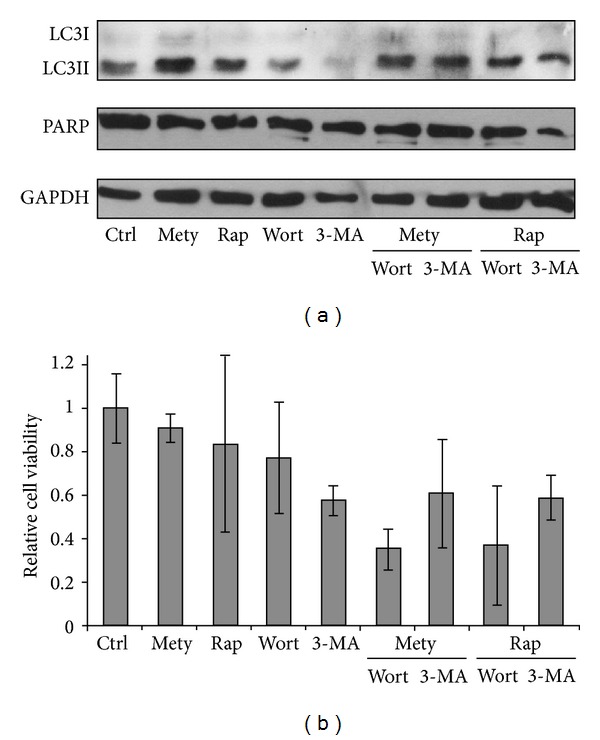
NADPH depletion and autophagy inhibition together promote cell death. HepG2 cells were treated with metyrapone (Mety—100 *μ*M), rapamycin (Rap—100 nM), Wortmannin (Wort—1 *μ*M), and 3-methyladenine (3-MA—10 mM) for 2 h at 37°C. Cells were also pretreated with Wort/3-MA for 2 h; then Mety/Rap was added for another 2 h. (a) Cell lysates were analysed by Western blot by immunoreacting with antibodies against LC3, PARP, and GAPDH, respectively. (b) Cell viability was followed by using Cell Titer-Blue Assay and the relative cell viability was represented after treatment.

To investigate further the general effect of luminal NADPH depletion, a viability assay was carried out and the viable cells were also counted (Figures 1(b) and 1(c)). These analyses showed—in accordance with previous findings [5]—that no cumulative cell death was present during the treatment at either metyrapone concentration for 1 hour (Figures 1(b) and 1(c)). These results suggest that high concentration of metyrapone results in drug-induced autophagy; however, this mechanism does not enhance “self-eating”-dependent cell death. To confirm the positive effect of metyrapone on autophagy induction a combined treatment was established with various autophagy inhibitors, such as 3-methyladenine and Wortmannin (Figure 2). Autophagy was less evident in both simultaneous treatments (metyrapone + 3-methyladenine and metyrapone + Wortmannin) and relative cell viability was also decreased (Figure 2). Cleaved PARP indicates apoptotic cell death at addition of Wortmannin even at combined treatment. Thus, NADPH depletion-induced autophagy showed similar characteristic to rapamycin treatment (Figure 2).

**Figure 3:**
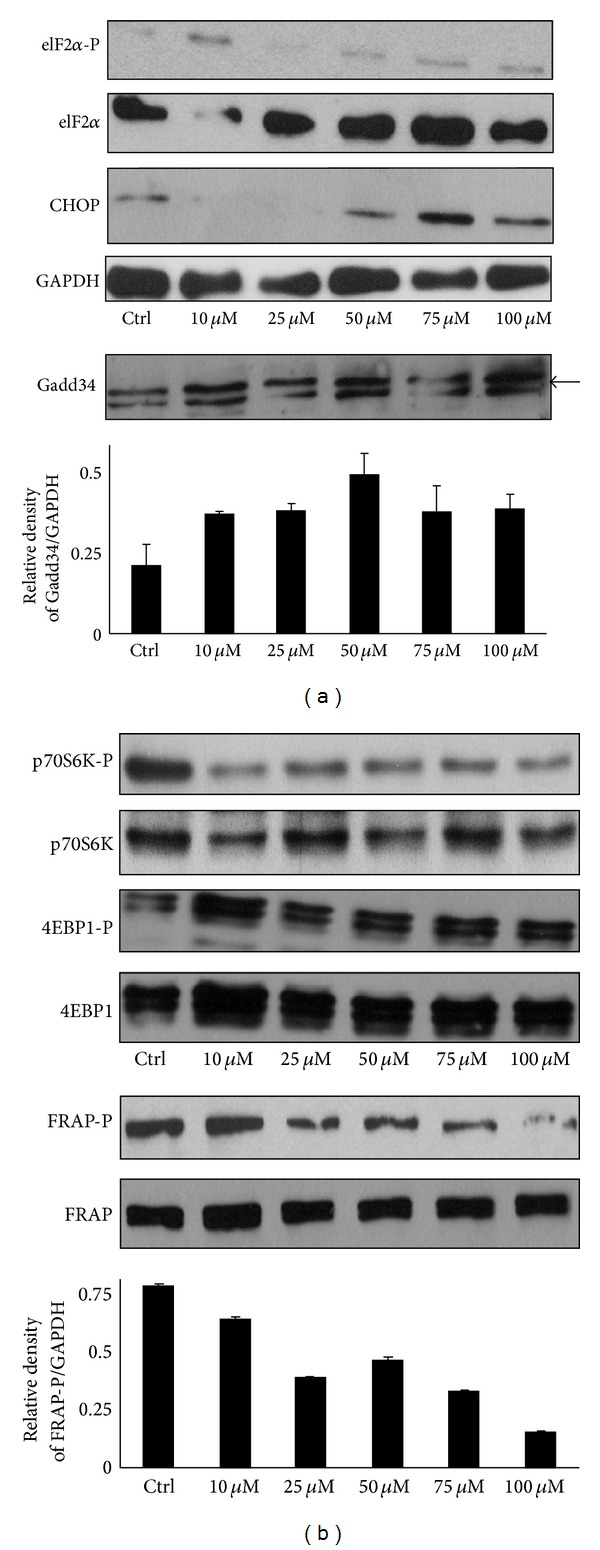
NADPH depletion induced by metyrapone enhances ER stress and influences the phosphorylation status of mTOR pathway proteins. HepG2 cells were treated with different amount of metyrapone (10 *μ*M–100 *μ*M) for 1 h at 37°C. (a) Cell lysates were analysed by Western blot by immunoreacting with antibodies against eIF2*α*-P, eIF2*α*, CHOP/Gadd153, GAPDH, and Gadd34, respectively. The relative density of Gadd34/GAPDH was plotted. (b) Cell lysates were analysed by Western blot by immunoreacting with antibodies against mTOR/FRAP-P, mTOR/FRAP, 4EBP1-P, 4EBP1, p70S6K-P, and p70S6K, respectively. The relative density of phosphorylated FRAP/GAPDH was represented.

Beside autophagy activation, a remarkable induction of Gadd34 and the slight but delayed activation of proapoptotic CHOP/Gadd153 could also be observed upon metyrapone addition (at concentrations >50 *μ*M) confirming ER stress (Figure 3(a)). eIF2*α* phosphorylation could not be observed after 1-hour treatment in accordance with the phosphatase activity of induced Gadd34 (Figure 3(a)).

As Gadd34 acts on mTOR/FRAP pathway negatively by dephosphorylating and activates the inhibitor TSC2 of mTOR/FRAP directly [20] we are interested in whether this starvation-controlled pathway is active or not during metyrapone treatment. Therefore, the phosphorylation of mTOR/FRAP kinase and its two well-known downstream targets (4EBP1 and p70S6K) was also followed by Western blotting (Figure 3(b)). Although Gadd34 gets activated and mTOR/FRAP becomes dephosphorylated at drug concentration >50 *μ*M, 4EBP1 is not shown any dephosphorylation after 1- hour treatment. These data suggest that mTOR pathway could not be fully inactived during redox imbalance of ER luminal pyridine nucleotides. Interestingly p70S6K kinase, the other target of mTOR/FRAP becomes almost completely dephosphorylated even after 1 hour treatment with 10 *μ*M metyrapone (Figure 3(b)) supposing that drug-induced luminal NADPH depletion of ER has actually some negative effect on mTOR/FRAP-dependent starvation pathway. 

### 3.2. The kinetic Profile of Metyrapone-Induced mTOR Inactivation

**Figure 4:**
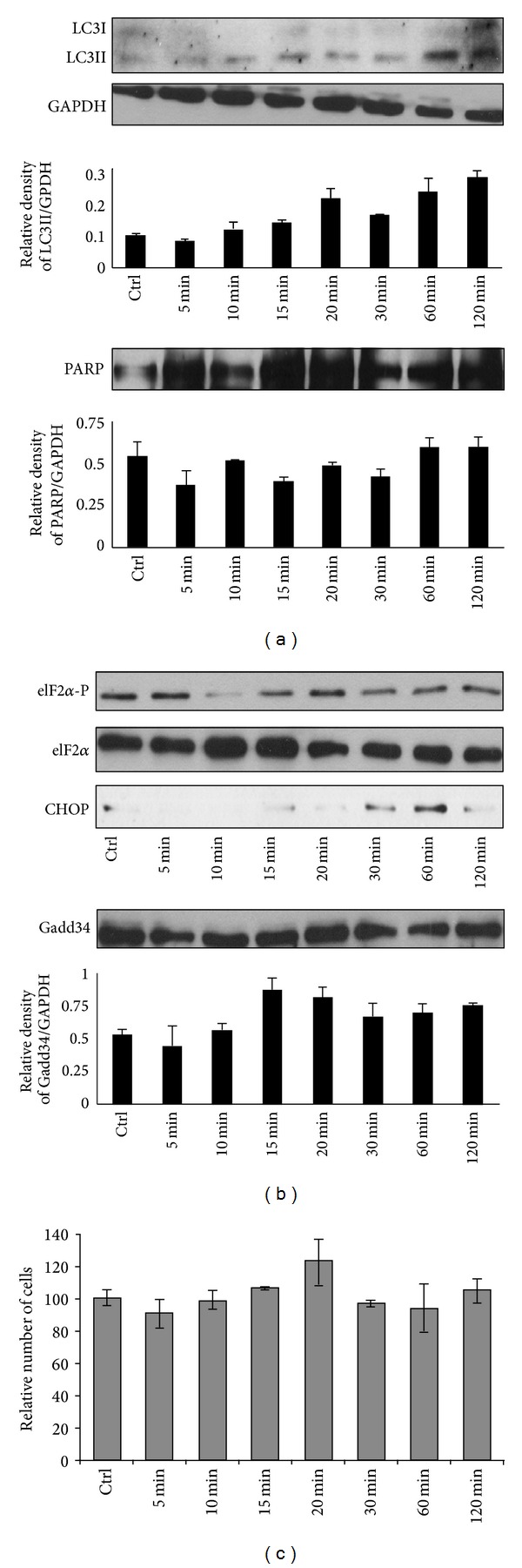
Time course of the effect of high concentration metyrapone on ER stress. HepG2 cells were treated with 100 *μ*M metyrapone for 5–120 min at 37°C. (a) Cell lysates were analysed by Western blot by immunoreacting with antibodies against LC3, PARP, and GAPDH, respectively. The relative density of both the lower band of LC3/GAPDH and PARP/GAPDH were represented. (b) Cell lysates were analysed by Western blot by immunoreacting with antibodies against ER stress markers, such as eIF2*α*-P, eIF2*α*, CHOP/Gadd153, and Gadd34, respectively. The relative density of Gadd34/GAPDH was represented. (c) Cell viability was assessed by counting the cells both permeable and non-permeable to trypan blue. About 5% of the cells were nonpermeable for trypan blue.

To get the time course of metyrapone treatment (100 *μ*M), its effects were followed in time. The level of LC3II was already elevated at 1 h and further increased at 2 h of incubation with 100 *μ*M metyrapone assuming autophagic process after 1-hour treatment (Figure 4(a)). Interestingly eIF2*α* phosphorylation and activity of both Gadd34 and CHOP/Gadd153 show only a transient peak (Figure 4(b)). Metyrapone treatment resulted in a stress-dependent UPR activation; however, the UPR elements completely disappeared after 120 min. These data also support the idea that autophagy activated by NADPH depletion promotes a cell survival process by adapting the cells to a tolerable stress condition. We claim that the metyrapone-induced ER stress is not severe enough to induce cell death. No PARP cleavage was observed during treatment supposing that apoptotic process remained inactive (Figure 4(a)). These results were also confirmed by carrying a viability assay, which did not show any cumulative cell death even after 2-hour long luminal redox imbalance (Figure 4(c)).

**Figure 5:**
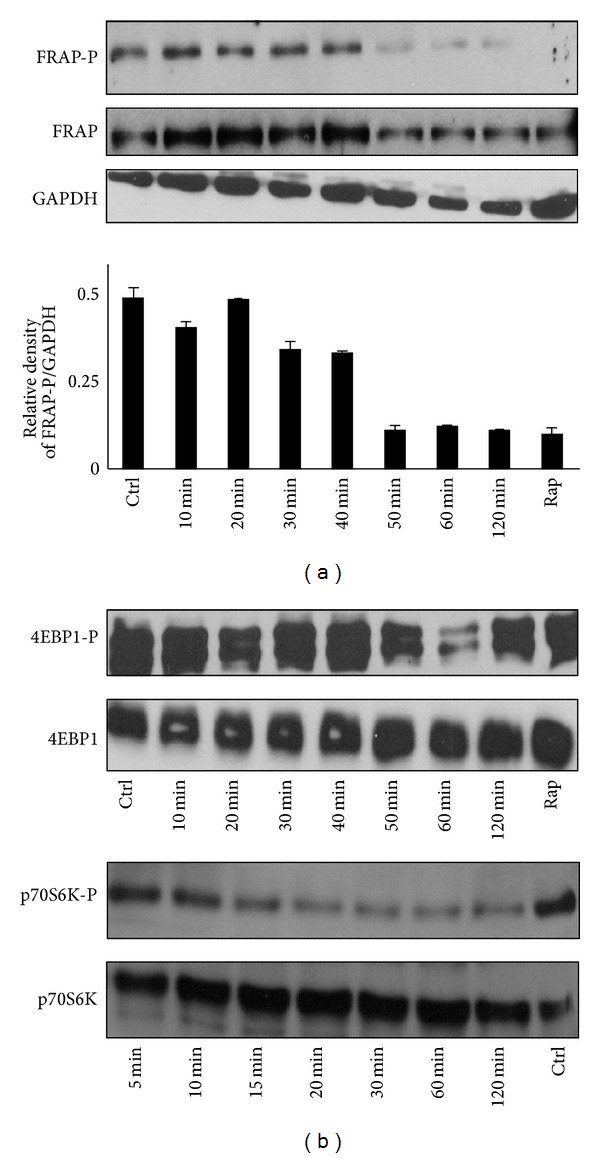
Time course of the effect of high concentration metyrapone on phosphorylation status of mTOR pathway proteins. HepG2 cells were treated with 100 *μ*M metyrapone for 5–120 min at 37°C. Each sample was analysed by Western blot by immunoreacting with antibodies against mTOR/FRAP-P, mTOR/FRAP, 4EBP1-P, 4EBP1, p70S6K-P, and p70S6K, respectively. The relative density of phosphorylated FRAP/GAPDH was represented. As positive control of autophagy cells were treated with 100 nM rapamycin for 1 hour at 37°C.

The time course of the phosphorylation of the key components of mTOR pathway was also examined. mTOR/FRAP gets dephosphorylated after 50–60 min, and this dephosphorylation became completed after 2 hours supposing the turning off of mTOR/FRAP pathway during treatment (Figure 5). While mTOR/FRAP getS dephosphorylated relatively late, the inactivation of p70S6K was observed after 15 min. Although both mTOR/FRAP and p70S6K kinase get dephosphorylated, mTOR/FRAP target 4EBP1 remains active and phosphorylated (Figure 5). This various activation profile of downstream targets of mTOR/FRAP suggests kinetic differences between them. It can also be possible that metyrapone treatment has an mTOR-independent effect on 4EBP1 and/or p70S6K phosphorylation/dephosphorylation.

**Figure 6:**
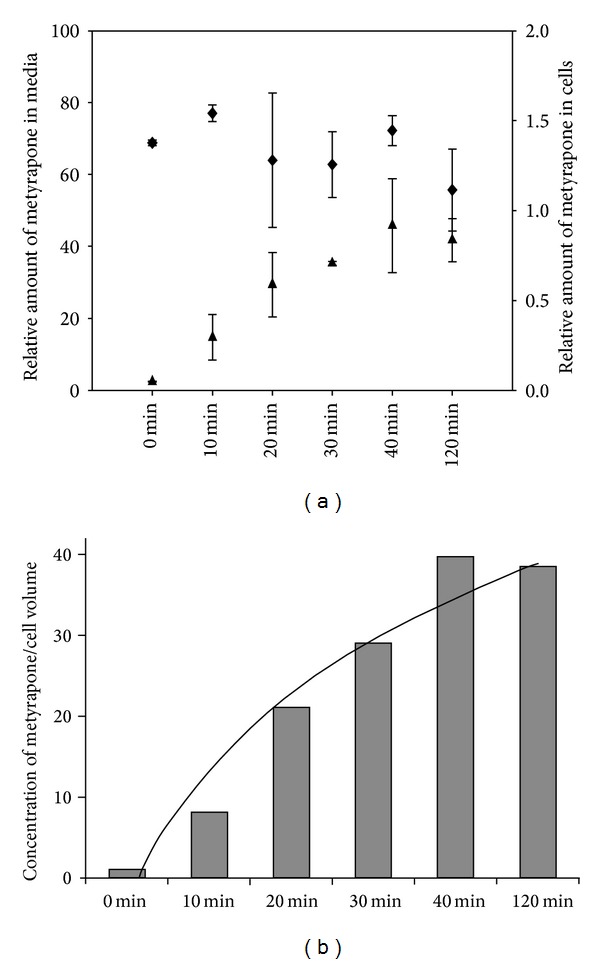
Metyrapone uptake of HepG2 cells. HepG2 cells were treated with 100 *μ*M metyrapone for 0–120 min at 37°C. (a) After cell lysis, cells metyrapone concentrations were measured by HPLC till 120 min upon the addition of metyrapone, and their relative amounts were calculated both in the media (black diamond) and the cells (black triangle). (b) Metyrapone concentration in cells referring to cell volume is plotted at each time point and a trend line was fitted (black hyperbolic line). For detailed description of HPLC method see Section 2.

To rule out the possibility that cells are able to adapt to metyrapone by pumping the drug out, its uptake was measured by HPLC. The increase of metyrapone concentration in the cells shows hyperbolic characteristic reaching a maximum level after 30 min and it was maintained on this level even after 120 min (Figures 6(a) and 6(b)). This picture shows that a constant metyrapone level was reached after 30 min in the cell, which was in agreement with the time course of metyrapone induced effects. 

### 3.3. Imbalance of ER Luminal NADPH/NADP^+^ Ratio and mTOR Inactivation by Silencing of G6PT and/or H6PDH

Although the role of metyrapone was already proved by imbalance the pyridine nucleotide level in luminal ER, the exact way of its action, and its targets have been not explored yet. We had to confirm that metyrapone is not able to act directly on one of the steps of mTOR pathway besides inducing the depletion of ER luminal NADPH. Therefore, the two key proteins of maintaining luminal NADPH level, G6PT, and H6PDH were silenced in HepG2 cells by transfection with the corresponding siRNA. G6PT is needed for glucose-6-phosphate uptake of the ER, while H6PDH transforms glucose-6-phosphate to 6-phosphogluconate by generating NADPH [24]. This transformation requires NADP^+^; therefore, silencing the expression of these two genes results in serious NADPH depletion in the ER lumen. 

**Figure 7:**
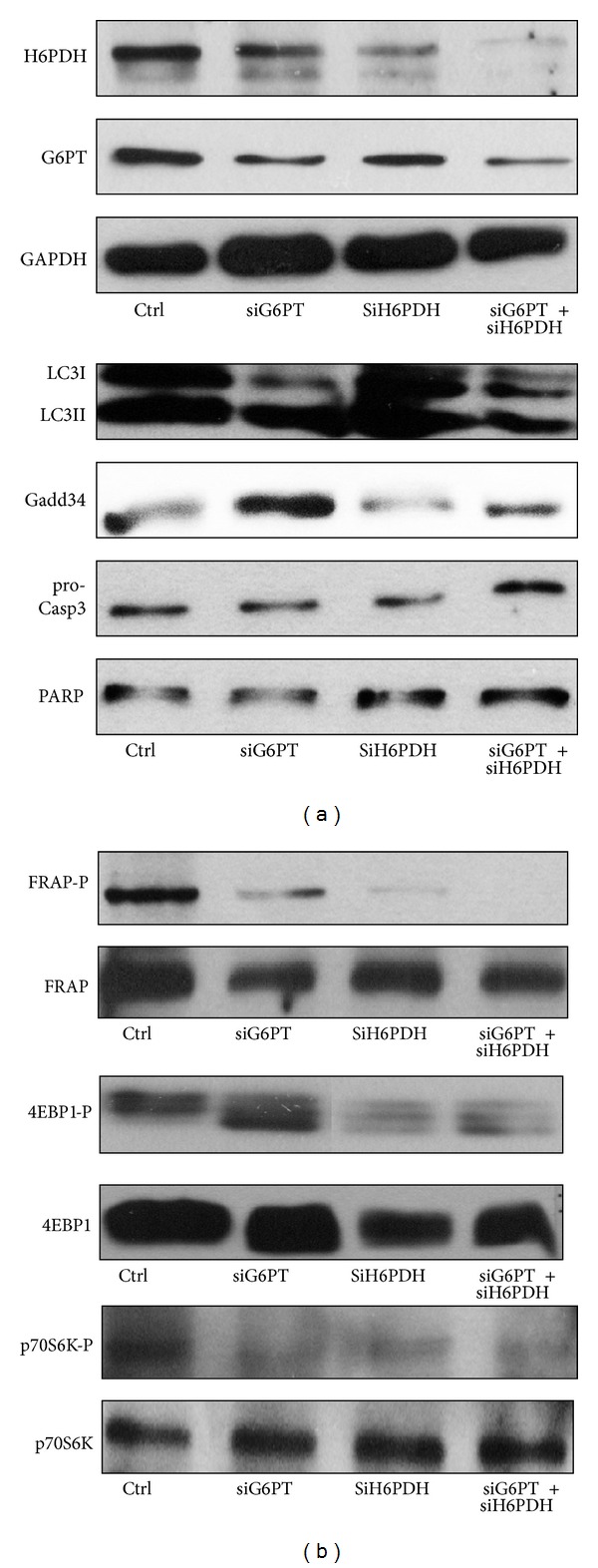
Transient knockdown of G6PT and/or H6PDH by siRNAs induces autophagy. HepG2 cells were treated with siRNA construct for G6PT and/or H6PDH. For description of this method see Section 2. Cells were lysed after 24 hours of silencing and samples were analysed by Western blot by immunoreacting with antibodies against G6PT, H6PDH, GAPDH, LC3, procaspase 3, PARP, Gadd34, mTOR/FRAP-P, mTOR/FRAP, 4EBP1-P, 4EBP1, p70S6K-P and p70S6K, respectively.

Transfection of HepG2 cells with siG6PT and/or siH6PDH reduced the level of G6PT and/or H6PDH dramatically (Figure 7). Both siG6PT and siH6PDH resulted in activation of autophagic event (Figure 7). This was demonstrated by the remarkable increase of LC3II level and intensive Gadd34 expression, while the cleavage of procaspase 3 and PARP was not observed. These data show that similarly to autophagy induced by metyrapone treatment, the survival process switches on during silencing of G6PT and/or H6PDH. Interestingly, the mTOR/FRAP target 4EBP1 gets fully dephosphorylated after silencing G6PT and/or H6PDH suggesting that imbalance of luminal NADPH/NADP^+^ ratio affects the mTOR/FRAP pathway negatively (Figure 7). Silencing of either G6PT or H6PDH, in agreement with our previous results [5], did not compromise the viability of the cells (data not shown).

## 4. Discussion

The present results show that depletion of ER NADPH either by the pharmacological agent metyrapone or by silencing of the key proteins of luminal NADPH generation switched on an autophagic mechanism with the incomplete inactivation of the mTOR pathway. 

We observed that high concentration of metyrapone was able to enhance the autophagy marker LC3II even after 1 h, suggesting that the imbalance of ER luminal pyridine nucleotides is alone sufficient to induce the “self-eating” mechanism (Figure 1). The ER-stress related autophagy inducer Gadd34 became active at relatively low concentration of metyrapone, while increased expression of the pro-apoptotic transcription factor CHOP/Gadd153 was detected only at high amount of drug (Figure 3(a)). Since this induction of CHOP/Gadd153 did not result in a decrease of cell viability in our experimental system (Figure 1), we suggested that autophagic response induced by NADPH depletion might have a role in cell survival. This observation was also confirmed by using autophagy inhibitors together with metyrapone (Figure 2). Interestingly, metyrapone after a short exposure (30 min) transiently increased the expression of transcription factor CHOP/Gadd153 following Gadd34 induction (Figure 4(b)), but cells remained viable (Figures 4(a) and 4(c)). These results suggest that first autophagy gets activated to turn on the “self-eating” mechanism to heal damages. Later apoptosis via CHOP tries to switch on but the stress level induced by NAPDH depletion is not high enough to develop the self-killing mechanism; therefore, CHOP/Gadd153 has only a transient peak. These results correspond to our recently published data where we claim that cellular stress induces autophagy-dependent survival immediately, while the stress level has to reach a critical threshold to promote apoptotic cell death [25].

Gadd34 has been shown to induce autophagy via the dephosphorylation of TSC2, which leads to the suppression of the mTOR pathway, a potent inhibitor of autophagy [21]. In agreement with the supposed role of Gadd34 in the regulation of mTOR pathway, a decreased phosphorylation of mTOR and p70S6K was also observed after longer incubations. However, the mTOR target 4EBP1 remains phosphorylated (Figure 5). This various activation profile of downstream targets of mTOR suggests kinetic differences between them. It can also be possible that metyrapone treatment has an mTOR-independent effect on 4EBP1 and/or p70S6K phosphorylation/dephosphorylation. These considerations are also supported by the observations that p70S6K dephosphorylation precedes that of mTOR (Figure 5) and requires lower metyrapone concentration (Figure 3(b)). Moreover, 4EBP1 dephosphorylation was evident upon silencing-based consumption of the luminal NADPH pool (Figure 7). It should be noted that although the downstream targets of mTOR (i.e., p70S6K and 4EBP1) are supposed to be regulated in line with each other, in some cases they are inhibited differently [26, 27]. 

The results gained by metyrapone were confirmed by silencing of the proteins responsible for generation ER luminal NADPH. Activation of the autophagic machinery was indicated by increased Gadd34 expression, LC3II formation, and dephosphorylation of 4EBP1 (Figure 7). 

In conclusion, the depletion of luminal NADPH in the ER (and/or the decreased NADPH/NADP^+^ ratio) results in ER stress followed by the activation of autophagic response. The scenario consists of the temporary increase of CHOP/Gadd153 without the alteration of cell viability. Thus, the NADPH depletion dependent ER stress unequivocally results in a physiological prosurvival response. Although the elements of mTOR pathway respond differently to imbalance of luminal pyridine nucleotides, these data suggested that the NADPH depletion-induced autophagosome formation has starvation-related phenotype. The findings underline the role of the redox state of ER luminal pyridine nucleotides (and the metabolic pathways affecting it) in the nutrient sensing of the cell [24].
